# 

**Published:** 1924-04

**Authors:** 


New Series. Vol. III. No. 31 (Vol. LXXIII. No. 1873). April, 1924. Monthly, Price 6d. (POST FREE 7½d.)

